# Do incident musculoskeletal complaints influence mortality? The Nord-Trøndelag Health study

**DOI:** 10.1371/journal.pone.0203925

**Published:** 2018-09-28

**Authors:** Anders Nikolai Åsberg, Knut Hagen, Lars Jacob Stovner, Ingrid Heuch, John-Anker Zwart, Bendik Slagsvold Winsvold

**Affiliations:** 1 Department of Neuroscience, NTNU Norwegian University of Science and Technology, Trondheim, Norway; 2 Norwegian Advisory Unit on Headache, St. Olavs University Hospital, Trondheim, Norway; 3 Department of Research, Innovation and Education, Division of Clinical Neuroscience, Oslo University Hospital, Oslo, Norway; 4 Institute of Clinical Medicine, University of Oslo, Oslo, Norway; 5 Department of Neurology and FORMI; Oslo University Hospital, Oslo, Norway; Monash University, AUSTRALIA

## Abstract

**Background:**

Musculoskeletal complaints (MSC) are common in the general population, causing a major disease burden to the individual and society. The association between MSC and mortality is still unclear. To our knowledge, no study has hitherto evaluated the association between MSC onset within the last month (incident MSC) on the one hand, and all-cause and cause-specific mortality on the other.

**Methods:**

This prospective population-based cohort study was done using data from the second Nord-Trøndelag Health Study (HUNT2) linked with data from a comprehensive national registry of cause of death. A total of 25,931 participants at risk for incident MSC were included. Hazard ratios (HR) of mortality were estimated for participants with incident MSC using Cox regression based on a mean of 14.1 years of follow-up.

**Results:**

Participants who reported incident MSC did not have an excess mortality compared to those with no MSC in the analyses of all-cause mortality (HR 0.99, 95% CI 0.89–1.10) and cause specific mortality. This was true also after adjustment for several potential confounding factors. No clear association between the number of MSC body sites and mortality was found.

**Conclusion:**

Incident MSC were not associated with an increased mortality, neither for all-cause mortality, nor cause-specific mortality.

## Introduction

Musculoskeletal complaints (MSC) are common in the general population, with a global prevalence of approximately 30% [1], which seems to be increasing [2–7]. Such complaints constitute a substantial individual and societal burden [3, 6, 8–10]. The leading cause of disability in 2013 were low back and neck pain in most western countries [11].

The relationship between MSC and mortality is still unclear. Several previous studies suggesting an increased mortality among those with MSC [12–17], whereas other studies have not confirmed this [18–23]. A review of existing literature from 2014 did not find a clear relationship between MSC and mortality, whereas an extended review from 2017 reported a statistically significant association between all-cause mortality and chronic widespread pain [15].

Most previous studies have focused on mortality caused by chronic and/or widespread MSC. To the best our knowledge, no previous studies have evaluated the relationship between new onset MSC and mortality. This is of relevance, because new onset MSC can be transformed to chronic MSC in about one out of three [24, 25], and because new onset MSC can generate fear of a serious illness. Furthermore, MSC predict long-term increase in general health care use [26]. Thus, evaluation of association between new onset MSC and mortality may have implication for future health care recommendations for MSC.

Very few population-based studies have evaluated the occurrence of new onset MSC. We have previously reported an annual incidence rate of MSC of 1.07 cases per person-year [27], nearly as high as upper respiratory infections in adults, with an incidence of 2–4 per person-year [28]. However, because information regarding previous episodes of MSC commonly is lacking, the incident MSC group will in practice consist of individual with previous experience of short-lasting MSC and individuals with first episode of MSC.

If MSC prove to be associated with mortality, the mechanisms for this are not clear. Some studies have suggested that the relationship between pain and mortality may be explained by other factors than the pain itself, e.g. physical inactivity[1, 29–32], functional limitation, and use of opioids. Incident MSC might be an underlying cause of physical inactivity, which in turn is associated to chronic diseases that increase mortality [33]. MSC have been associated to perceived reduction of physical ability [34], and reduced physical ability have been associated with mortality [35]. A recent study reported functional limitation and physical inactivity to be a strong mediating factor in the relationship between troubling pain and mortality [36]. Furthermore, a link between mortality and prescription of opioids in patients with chronic noncancer pain has been found [37]. In addition, when investigating the association between MSC and mortality, it is imported to consider potential known confounding factors like low education level and smoking [1, 29–32], factors that also are associated with cardiovascular disease (CVD) and cancer [33, 38].

This study aimed to evaluate the association between incident MSC and all-cause mortality and cause-specific mortality. We used data from a large population-based follow-up study with comprehensive health information, allowing adjustment for potential confounding factors.

## Methods

### Study area

The second Nord-Trøndelag Health Survey (HUNT2) was undertaken between August 1995 and June 1997, in the county of Nord-Trøndelag in central Norway. Here, the population is scattered and no city has more than 21,000 residents [39]. The population is stable and homogenous, and fairly representative of the general Norwegian population, although average income and education levels are a little lower than the country’s average [39].

### Procedure

For the survey, all inhabitants aged 20 years or older were invited to participate. Participants had to complete two extensive questionnaires at different time points (Q1 and Q2) and attend a medical examination. The examination included having blood samples taken, and measurements of height, weight and blood pressure.

The invitation was sent by mail, and the first questionnaire (Q1) was attached and to be completed at home the previous week or several weeks before the health examination. The second questionnaire (Q2) was filled in at home after the health examination and returned by mail. The median number of days between the health examination, including the return of Q1, and the day Q2 was filled in at home was 2 days (range 0–30 days). Further details regarding HUNT2 is available elsewhere [39]. The exact time interval between answering first Q1, and then Q2 at home after the health examination, was not possible to estimate as we do not have information regarding the exact time when the participants answered Q1 after receiving it by mail, but for the majority of participants the interval was most likely 2–4 weeks.

### Definition of incident MSC

Q1 included a screening question for chronic MSC: “Have you during the last year continuously for at least 3 months had pain and/or stiffness in muscles and joints?” Participants who answered “no” to this screening question were defined as “population at risk” for incident MSC and included in the present study.

**Fig 1:**
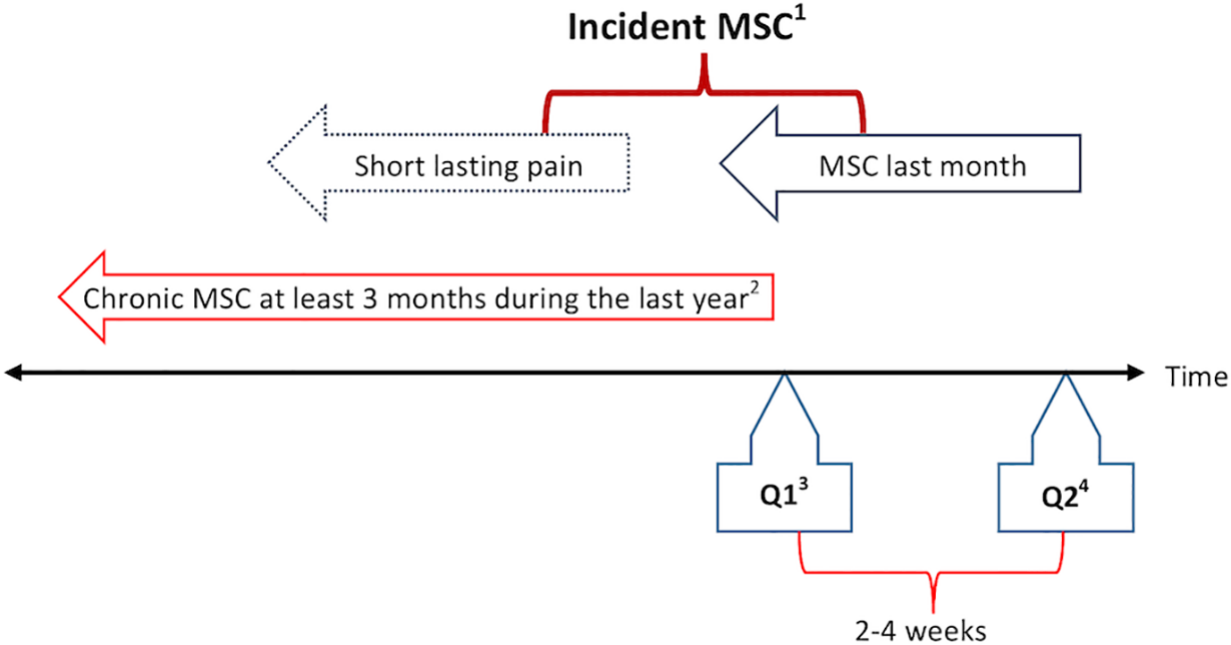
^1^MSC = Musculoskeletal complaints. ^2^Excluded from the analyses. ^3^Q1 = The first questionnaire, received by mail and completed at home. ^4^Q2 = The second questionnaire, received after the health examination.

Q2, completed at home approximately 2–4 weeks after Q1, included the screening question for incident MSC “Have you during the last month had complaints (pain and/or stiffness) from muscles and/or joints?” Subjects who answered “yes” constituted the group with incident MSC. We had no information regarding previous episodes lasting maximum 1–2 months with MSC during the last year. Thus, these persons were included in the group with incident (Fig 1).

Individuals who confirmed MSC during the last month were also asked to indicate the number of days with MSC, and tick off 1 or several of the following 9 areas of the body; neck, shoulders, elbows, wrist/hands, upper back, lower back, hips, knees, and/or ankles/feet. Based on the number of days with MSC, subjects were categorized as either ≥ 15 days or < 15 days, and based on the number of body sites, they were categorized as 1–2 or > 2 body sites with MSC. To evaluate the impact of incident MSC on daily life, participants were asked the questions: “Within the last month, have these complaints hindered you in completing daily tasks in your work?” and “Within the last month, have these complaints hindered you in completing daily tasks in outside work?”. Subjects who answered yes to both questions constituted the group with MSC interfering with daily life.

### Cause of death and follow-up

All inhabitants of Norway are given an 11-digit person identification number upon birth. This allows for a complete follow-up on mortality in the country, as all deaths are required to be reported by a physician to the National Cause of Death Registry with a cause of death diagnosis [40]. Autopsy has been performed in about 4% of cases [41]. Data on the cause and date of death were obtained from the national database for all participants from the date of attendance in HUNT2 until December 2011. In the present study, the cause of death was categorized into three groups; cardiovascular death (International Statistical Classification of Diseases and Related Health Problems, 10th revision [ICD-10 code I00-99]), death from cancer (ICD-10 code C00-C97), and other types of death (all other ICD-10 codes).

### Study population

Among 92,936 invited to HUNT2, 65,026 (70%) answered Q1, of which 49,746 also answered the MSC question in Q2.

### Potential confounders

HUNT2 included a wide range of health-related information, and several variables, in particular age and gender, have previously been identified to be associated with incident MSC [27].

Several other potential confounding variables associated with mortality were also constructed and included in the analysis. Smoking was categorized as “current daily smoking”, “previous daily smoking”, or “never daily smoking”. Physical activity was categorized on the basis of the amount of hard physical activity per week: High (3 or more hours of hard physical activity per week, i.e. being out of breath and/or sweating), medium (1–2 hours of hard physical activity per week), or low (less than 1 hour of hard physical activity per week or any form of light physical activity) [30]. Education was categorized as less than 10 years, 10–12 years or more than 12 years. Body mass index (BMI) was categorized as less than 25.0 kg/m^2^, 25.0 to 29.9 kg/m^2^ or more than or equal to 30.0 kg/m^2^. The use of alcohol was categorized as being either abstainer or not. The Hospital Anxiety and Depression Scale (HADS) was used to assess anxiety and depression [42]. Metabolic syndrome and self-reported CVD was included as present or not. Systolic blood pressure was included as a continuous variable.

### Statistical analysis

Differences in means of continuous variables between the group with incident MSC and the group without incident MSC were tested with the Student’s t-test and differences in proportions by the chi-square-test. To examine the association between incident MSC and mortality, the Cox proportional hazard regression was used to estimate hazard ratio (HR) of death. Furthermore, to evaluate the impact of number of body sites with pain, individuals with incident MSC were subdivided into two groups, 1–2 MSC body sites, and > 2 body sites. All analyses were initially done with adjustment for age (as a continuous variable) and sex (model 1). They were then repeated with adjustment for several potential confounders in model 2; smoking (3 categories), physical activity (3 categories), education (3 categories), total HADS (3 categories), BMI (3 categories), alcohol use (2 categories), self-reported CVD (yes/no), metabolic syndrome (yes/no) and systolic blood pressure (continuous variable). Subjects with an incomplete data set were included as a separate missing category to reduce the impact of response bias. To test for possible interactions between age and sex and incident MSC in the prediction of mortality, we added a product term (e.g. gender and incident MSC) in the Cox regression model. Statistical significance was set at p < 0.05. IBM SPSS version 24 was used for all analyses.

### Ethics

HUNT2 was approved by the Norwegian National Data Inspectorate and the study was also approved by the regional committee for medical and health research ethics (REC). This study was also approved by the REC.

## Results

**Fig 2:**
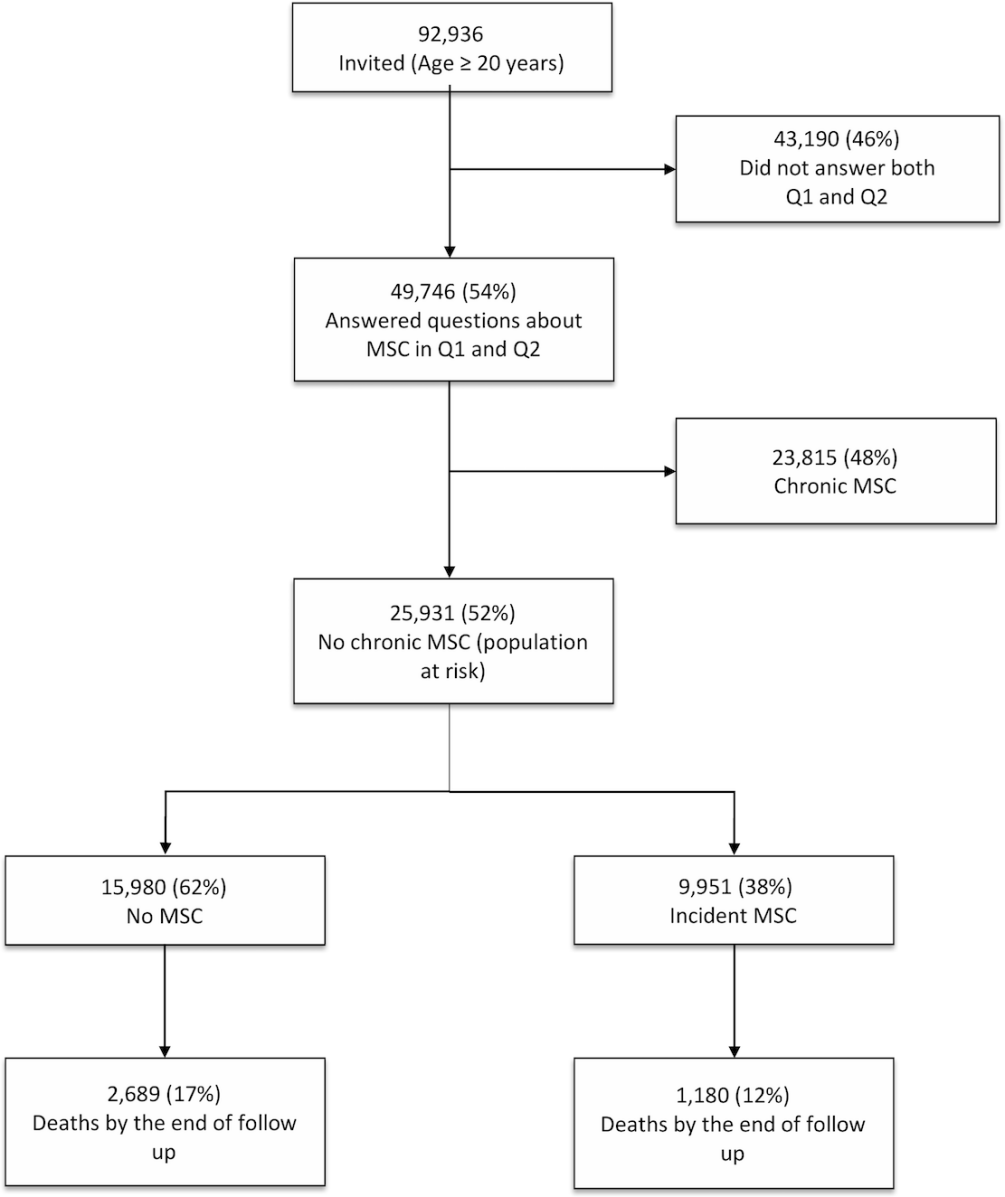
Flowchart of the study population.

Among the 49,746 who answered the question about MSC in Q1 and Q2, 25,931 subjects reported no chronic MSC in Q1 and were defined as population at risk of incident MSC (Fig 2). Among these, 9,951 (38%) reported MSC within the last month. Of these, 9,056 (91%) reported body site for MSC. Table 1 presents the baseline distribution of the clinical and demographic characteristics. Those who reported incident MSC were more often female, younger, more likely to smoke and less physically active. This group also had a lower mean systolic blood pressure and a lower proportion who reported CVD compared to those with no MSC.

**Table 1:** Demographic and clinical characteristics related to incident MSC status in HUNT2.

Variable	No incident MSC	Incident MSCs
Participants, no	15,980	9,951
Mean age at HUNT2, years (SD)^1^	47.1 (18.0)	44.9 (16.1)*
Person-years^2^, no	228806	145922
Gender, female (%)	48.5	53.2*
Education > 12 years (%) (missing^a^ = 607)	24.4	24.9*
Self-reported CVD^3^ (%)	7.8	6.9*
Mean BMI^4^ (SD), kg/m^2^	25.8 (3.8)	25.9 (3.9)
Physical activity, low (%) (missing^a^ = 1289)	61.9	64.7*
Daily smoking (%) (missing^a^ = 289)	23.6	26.8*
Alcohol abstainer (%) (missing^a^ = 340)	10.8	8.5*
Total HADS^5^ (SD) (missing^a^ = 581)	6.0 (4.7)	7.1 (5.0)*
Metabolic syndrome (%)	15.2	14.4
Mean systolic BP^6^ (SD), mmHg	137.3 (21.6)	134.0 (20.0)*
Number of death after HUNT2	2689	1180
Mean age after HUNT2, years (SD)^1^	79.3 (11.9)	77.9 (12.4)

^1^SD = Standard Deviation

^2^Person-year: The sum of time each participants contributed with from time of inclusion to death or end of follow up in december 2011.

^3^CVD = Cardiovascular Disease

^4^BMI = Body Mass Index

^5^HADS = Hospital Anxiety and Depression Scale

^6^BP = Blood Pressure.

^a^Missing values are given as numbers of individuals when not zero.

*Significantly different from those without incident MSC (p<0.05)

Total follow-up time amounted to 374,728 person years, during which time a total of 3860 died. CVD accounted for 1,589 deaths, while 1,142 were due to cancer and 1,138 were due to other causes than CVD and cancer.

There was no evidence that the association between incident MSC and mortality was modified by gender (p_interaction_ = 0.63) or age (p_interaction_ = 0.68). Thus, all results are presented for men and women combined.

The analysis of mortality with adjustments for age and sex (model 1) showed no excess mortality for either all-cause mortality, mortality of CVD, cancer mortality or mortality of all other causes (Table 2). This was true for both the group with complaints < 15 days and ≥ 15 days. When the analyses were repeated with additional adjustments for education, smoking, self-reported CVD, alcohol, BMI, systolic BP, metabolic syndrome, physical activity and HADS (model 2), the HRs decreased slightly, and no significant excess or reduced mortality was found for any of the categories of mortality (Table 2).

**Table 2:** Mortality among participants with incident musculoskeletal complaints (MSC) within the last month compared to those without incident MSC. n = 25931.

	Total	Mortality of all causes		CVD^1^ mortality		Mortality of cancer		Mortality of all other causes^2^	
Variable		n	HR (95%CI)	n	HR (95%CI)	n	HR (95%CI)	n	HR (95%CI)
Adjustment model 1^3^									
No MSC	15980	2689	1.00 Reference	1114	1.00 Reference	768	1.00 Reference	807	1.00 Reference
Incident MSC, < 15 days^5^ (%)	7634 (29,4)	905	0.99 (0.92–1.08)	365	1.01 (0.89–1.13)	289	1.03 (0.90–1.18)	251	0.94 (0.81–1.08)
Incident MSC, ≥ 15 days^6^ (%)	2317 (8,9)	275	0.98 (0.87–1.11)	110	0.95 (0.78–1.15)	85	0.95 (0.76–1.19)	80	0.97 (0.77–1.22)
1–2 body sites (%)	6313 (24,3)	753	0.96 (0.89–1.05)	287	0.97 (0.85–1.10)	232	0.97 (0.83–1.12)	234	0.99 (0.86–1.15)
>2 body sites (%)	2743 (10,6)	274	1.04 (0.92–1.18)	124	1.08 (0.90–1.31)	94	1.05 (0.85–1.31)	56	0.74 (0.56–0.97)
MSC interfering daily activities^7^ (%)	747 (2,9)	42	1.01 (0.74–1.37)	14	1.03 (0.61–1.76)	16	0.95 (0.56–1.57)	12	1.00 (0.56–1.79)
Adjustment model 2^4^									
No MSC	15980	2689	1.00 Reference	1114	1.00 Reference	768	1.00 Reference	807	1.00 Reference
Incident MSC, < 15 days	7634	905	0.98 (0.90–1.05)	365	0.96 (0.85–1.08)	289	1.01 (0.88–1.16)	251	0.94 (0.81–1.08)
Incident MSC, ≥ 15 days	2317	275	0.91 (0.81–1.04)	110	0.84 (0.69–1.03)	85	0.87 (0.69–1.09)	80	0.92 (0.73–1.16)
1–2 body sites	6313	753	0.95 (0.87–1.03)	287	0.91 (0.80–1.04)	232	0.94 (0.81–1.09)	234	0.98 (0.85–1.14)
> 2 body sites	2743	274	0.97 (0.85–1.10)	124	0.97 (0.81–1.18)	94	0.98 (0.79–1.22)	56	0.71 (0.54–0.93)
MSC interfering daily activities^5^	747	42	0.93 (0.68–1.27)	14	0.97 (0.56–1.66)	16	0.83 (0.50–1.37)	12	1.00 (0.56–1.79)

1. CVD = Cardiovascular disease

2. Other causes = Death by other causes than cancer or cardiovascular disease.

3. Model 1: Adjusted for age and gender.

4. Model 2: Adjusted for age, gender, education, smoking, self-reported cardiovascular disease, alcohol, BMI, systolic BP, metabolic syndrome, physical activity and HADS.

5. Subjects who reported experiencing complaints less than 15 days during the last month

6. Subjects who reported experiencing complaints 15 days or more during the last month

7. Reporting that MSC hindered completing daily tasks during and outside work

Further, no excess mortality was found among subjects with MSC in 1–2 body sites or > 2 body sites, neither with adjustment for age and sex nor with adjustment for all the other potential confounders (Table 2). In fact, for > 2 body sites, the mortality of “all other causes” were actually slightly lower (HR 0.71, 95% CI 0.54–0.91).

A total of 747 participants reported that the MSC hindered them in completing daily tasks at work and elsewhere. In this group, the unadjusted analyses did not show an excess mortality, neither for “all cause” mortality (HR 1.01, 95% CI 0.74–1.37), mortality of CVD (HR 1.03, 95% CI 0.61–1.76), cancer mortality (HR 0.95, 95% CI 0.56–1.56) or mortality of all other causes (HR 1.00, 95% CI 0.56–1.79) (Table 2). When adjusting for the additional potential confounding factors, results remained statistically insignificant (Table 2).

## Discussion

In this large prospective cohort study, we found no significant association between incident MSC and all-cause mortality, mortality for CVD, cancer mortality or mortality for all other causes. This held true for both partly adjusted and fully adjusted analyses. The only exception was a lower mortality of “all other causes” in those with MSC in > 2 body sites. We cannot see any obvious reason for this, and since we made no corrections for multiple comparisons we believe it to be an incidental finding.

The group with incident MSC had declared that they were without chronic MSC in Q1. When responding to Q2 some weeks or days later they nevertheless reported MSC during the last month. This group, constituting more than a third of the population at risk (9,951 of 25,931), presumably consisted of persons who were prone to have shortlasting MSC. However, shortlasting MSC are very common in the general population as they come and go. Hence, it cannot be known for certain whether a new interrogation some time later would have defined more or less the same or quite a different group of individuals as having incident MSC. Nevertheless, since no difference in mortality was found in those with and without incident MSC, there is no reason to believe that this would have changed the conclusion. Indeed, it would have been surprising to find an increased mortality in persons with incident MSC when no increase was found in those with chronic MSC [20].

### Study strengths and limitations

The major strengths of this study are the longitudinal population-based cohort design with a large number of participants, a wide age range, and the long follow-up time of 14 years. Furthermore, using HUNT2 data coupled with data from the National Cause of Death Registry allowed for a complete follow-up on mortality. In addition, the extensive questionnaire made adjustment for several potential confounders possible. The use of questionnaire-based information about MSC might bias the results, but the reliability of the screening question on MSC in HUNT2 has earlier been reported to be satisfactory [2].

Several study limitations should also be considered. Firstly, results should be generalized with caution, because only 56% of the invited answered the questions about MSC in both Q1 and Q2. A study of 326 non-participants revealed that 10% reported immobilization by disease as a reason for not attending, and 10% reported follow-up by a physician or hospital as a reason for not attending [39]. It is unknown whether a participation rate of 100% would have changed the results, and if so, in what direction. Secondly, a low number of events in some of the subgroups analyses make the results less certain. Thirdly, the finding of reduced mortality might also be due to overadjustment, which tend to be an issue in epidemiological studies [43]. However, the absolute change in the estimated HRs from the analyses with adjustment for age and sex to the analyses with adjustment for several potential confounders was low, and findings were not statistically significant except for one subgroup. Fourthly, unfortunately no pain severity measure of MSC was included in the questionnaire. This could have been of relevance, since a subgroup with severe pain was found to be associated with all-cause mortality, where no such association was found when pain severity was not considered [17]. Fifthly, we did not have information regarding previous short-lasting episodes of MSC during the last year. Thus, it should be emphasized the group of incident MSC consisted of those with new onset MSC and those with recurrence of previous short-lasting MSC. Sixthly, we had no information about prescription of opioids. This would have been of interest, because increased mortality has been reported in persons with chronic noncancer pain using opioids [37]. Finally, the medical examination did not include diagnostic procedures for MSC, and self-reported questionnaire information regarding e.g. rheumatic diseases is not useful [44].

### Comparisons with other studies

Based on data from HUNT2, we have previously reported no association between chronic MSC and chronic widespread MSC and mortality [20], which is in accordance with a later, large population-based study from Tromsø, Northern Norway [21].

To our knowledge, this is the first study evaluating the impact of incident MSC on mortality. Accordingly, our results cannot be directly compared with the other studies reporting mortality in CMSC. However, the association between body pain sites and mortality has been evaluated in two different population-based studies from England with divergent findings [14, 23]. In the most recent study, using two different population cohorts, no association between the number of body pain sites and mortality was found [23], whereas the other study demonstrated an association between the number of pain sites and mortality of cancer [14].

In the present study, we did not find any relationship between incident MSC interfering with daily tasks during or outside work, and mortality. In contrast, a statistically significant association between pain interference on normal work and mortality was found in the recently published study from England [23]. The authors concluded that it was the impact rather than the mere presence of pain that explained the relationship between pain and mortality [23]. The reason for the divergent findings on pain interference with daily activity and mortality in that study and our study is unclear, but could be related to differences in study methodology (questions regarding pain interference with normal work were not identical), or to differences in the working life and type of work between England and Norway. Finally, our study might have less statistical power to detect a slight increase in mortality among those who reported MSC to interfere with daily activities the, due to few deaths in this group (n = 42). On the other hand, no increased mortality was found in the total group with incident MSC, which included a large number of deaths (n = 1180). In general, it is difficult to explain the divergence between the studies evaluating the association between mortality and MSC. In future studies, the importance of homogeneity between studies should be given priority, as this would make it easier to compare studies evaluating the impact of MSC on mortality.

In summary, no excess mortality was found. Even though we did not find an excess mortality among individuals with incident MSC, such health complaints are nevertheless major burdens to both the society and the individual. Therefore, future studies should evaluate how lifestyle factors and other factors impact the risk of developing MSC, in order to prevent the condition from developing.

## Conclusion

In this large, prospective population based cohort study we found that participants with incident MSC did not experience an excess mortality compared to those with no MSC.
